# Real-World effectiveness of eculizumab in atypical hemolytic uremic syndrome: a retrospective study from Western China

**DOI:** 10.3389/fendo.2025.1568082

**Published:** 2025-06-27

**Authors:** Yun Hu, Yanyan Zhang, Wei Wang, Guisen Li, Shasha Chen

**Affiliations:** ^1^ Department of Gastroenterology, Sichuan Academy of Medical Sciences & Sichuan Provincial People’s Hospital, Chengdu, China; ^2^ University of Electronic Science and Technology of China, Department of Clinical Medicine, Chengdu, China; ^3^ Department of Nephrology, Sichuan Academy of Medical Sciences & Sichuan Provincial People’s Hospital, Chengdu, China

**Keywords:** atypical hemolytic uremic syndrome, eculizumab, retrospective study, China, efficiency, safety

## Abstract

**Objective:**

To evaluate the real-world efficacy and safety of eculizumab in atypical hemolytic uremic syndrome (aHUS) within a Western Chinese cohort, with emphasis on treatment initiation timing and renal outcomes.

**Methods:**

We conducted a retrospective analysis of 17 aHUS patients treated at Sichuan Provincial People’s Hospital, focusing on the relationship between treatment timing and clinical prognosis. To evaluate timing effects, patients were categorized as Early Initiators (treatment within 7 days of symptom onset, n=9) versus Delayed Initiators (treatment beyond 7 days, n=8) The main outcome measures included hematological parameters, renal function, and adverse events.

**Results:**

The cohort (n=17, 76.5% female, mean age 43.2 ± 20.0 years) demonstrated significant improvements post-eculizumab: creatinine decreased from 647.0 (439.0, 915.0) to 198.5 (86.5, 749.5) μmol/L, eGFR increased from 8.0 (5.0, 11.0) to 22.1 (6.4, 55.1) mL/min/1.73m², platelets rose from 75 ± 11 to 143 ± 33×10^9^/L, and LDH declined from 787.5±908.0 to 232.8 ± 70.0 U/L (all P<0.001). Early treatment initiation (≤7 days, n=9) yielded superior outcomes versus delayed (>7 days, n=8): higher renal remission (88.9% vs 12.5%, P=0.003), hematological remission (100% vs 12.5%, P<0.001), and reduced dialysis dependence (0% vs 87.5%, P<0.001), with greater ΔeGFR (+(19.5 ± 3.1) vs +(2.3 ± 1.7 )mL/min/1.73m^2^, P=0.016) and Δplatelets (+(67.8 ± 9.8) vs +(19.3 ± 7.2)×10^9^/L, P=0.007). Renal survival favored early treatment (log-rank P<0.001), though residual renal impairment persisted. Two non-meningococcal bloodstream infections resolved with antibiotics.

**Conclusion:**

Our findings provide the first Chinese evidence supporting early eculizumab initiation (≤7 days post-symptom onset) significantly improves hematological/renal outcomes and reduces dialysis dependence in Chinese aHUS patients. Despite residual renal impairment, prompt complement blockade mitigates ESRD risk, supporting time-sensitive intervention in resource-limited settings.

## 1 Introduction

Atypical Hemolytic Uremic Syndrome (aHUS) is a rare and devastating condition that profoundly impacts both renal function and the hematologic system, significantly diminishing the quality of life for affected individuals and imposing a considerable economic burden on society (1–4). The incidence of aHUS is approximately 2 per 1,000,000 in adults and around 3.3 per 1,000,000 in children under the age of 18 (2). Prior to the introduction of complement inhibitors, management in Chinese centers heavily relied on plasma exchange and immunosuppression, which often yielded limited renal recovery and high complication rates (5); however, these approaches are characterized by inconsistent efficacy and the potential for adverse effects. Consequently, there is an urgent need to explore novel therapeutic modalities and rigorously assess their capacity to enhance patient outcomes.

Since its approval in 2007, the efficacy and safety of eculizumab have been validated across diverse populations. Landmark trials, such as the phase 3 study by Legendre et al (6), demonstrated its ability to halt renal damage in aHUS, with long-term safety confirmed in a 2-year extension study (7). Real-world evidence further supports these findings: Fakhouri et al (8) reported sustained hematologic remission in adults, while Greenbaum et al (9) highlighted its safety in pediatric cohorts. Recent observational studies, including a Japanese nationwide analysis (10), emphasize its role in improving early diagnosis and treatment adherence. Emerging data underscore the critical importance of timing, as initiating eculizumab within 7 days of symptom onset correlates with superior renal survival (11), particularly in patients with genetic predispositions (12). It was not until the end of 2024 that Eculizumab became available in China. Nevertheless, studies have not comprehensively evaluated the long-term safety and efficacy of Eculizumab within diverse patient cohorts in China, thereby constraining the generalizability of their clinical application. This retrospective analysis represents the first evaluation of the therapeutic efficacy and prognostic significance of Eculizumab within a Chinese cohort of patients with atypical Hemolytic Uremic Syndrome (aHUS).

## 2 Materials and methods

### 2.1 Study population and eligibility criteria

This retrospective cohort analysis enrolled adults (≥18 years) with thrombotic microangiopathy (TMA) treated with eculizumab at a tertiary care center in Southwest China (Sichuan Provincial People’s Hospital) between 2022-2024. Participants were required to meet diagnostic thresholds for TMA: (1) thrombocytopenia (platelets <150×10^9^/L), (2) microangiopathic hemolysis (hemoglobin ≤LLN, schistocytes ≥1%, negative Coomb’s), (3) elevated LDH (>1.5×the upper limit of normal (ULN); reference: 120-246 U/L), and (4) serum creatinine exceeding age-specific 97th percentiles. We excluded patients with: (1) congenital TMA disorders (ADAMTS-13 activity <10% or PLASMIC score ≥5 when unavailable) (Definitive ADAMTS-13 activity results were obtained for all enrolled patients within 2-5 days. Patients with ADAMTS-13 activity <10% were subsequently excluded, providing a more robust confirmation of non-TTP status than relying solely on the PLASMIC score.) (2) infectious etiologies (Shiga toxin/EHEC positivity), or (3) confirmed complement gene mutations indicative of primary aHUS.

Patients were grouped into Group 1 (≤7 days, n=9) and Group 2 (>7 days, n=8) based on Based on the Interval from Symptom Onset to Eculizumab Initiation. The 7-day threshold aligns with established efficacy windows [11] and regional diagnostic challenges. This investigation followed medical ethics guidelines stated in the Declaration of Helsinki and was authorized by the Ethics Committee of Sichuan Provincial People’s Hospital (Ethical Review Research No. 2024685). All participants were informed about the study before giving their consent to participate.

### 2.2 Dosages

Consistent with global protocols, patients received an initial eculizumab induction regimen (900 mg weekly ×4 doses, followed by 1,200 mg maintenance every 2 weeks). All patients received meningococcal vaccination ≥2 weeks pre-eculizumab and penicillin prophylaxis (azithromycin if allergic). Concomitant antibiotic prophylaxis (penicillin derivatives or, if allergic, azithromycin) was administered at treatment start to mitigate infection risk until adequate vaccine immunity developed for the minority of patients where vaccination occurred less than 2 weeks prior to eculizumab initiation (6).

### 2.3 Data collection

Baseline demographics, longitudinal laboratory indices (including hemoglobin, platelet counts, serum creatinine, and eGFR trajectories), therapeutic protocols, and safety profiles were systematically analyzed. Renal biopsies were conducted in 8 patients (53.3%) to confirm TMA diagnosis, with histopathological assessments documenting characteristic microangiopathic features: glomerular endothelial swelling, arteriolar “onion-skin” hyperplasia, sclerosed glomeruli counts, and tubulointerstitial injury severity (atrophy/fibrosis index). The majority (5/8) underwent comprehensive immunofluorescence profiling for IgA, IgM, IgG, C3, C4, and C1q deposition patterns, while three specimens lacked sufficient tissue for full antibody panels.

### 2.4 Definitions

Hematological remission required sustained platelet count >150×10^9^/L and hemoglobin ≥ sex-specific lower limit normal without transfusions for ≥4 weeks. Renal remission was classified as: (1) complete - eGFR recovery to within 10% of pre-morbid baseline; or (2) partial - ≥50% improvement from nadir eGFR. These standardized definitions align with endpoints from key clinical trials and KDIGO recommendations (6).

### 2.5 Statistical analysis

Baseline and longitudinal variables were analyzed through a dual approach: descriptive statistics summarized patient profiles using means ± SD or medians (IQR) for continuous measures and frequencies (%) for categorical data. Comparative analyses of pre-post treatment biomarkers employed distribution-appropriate tests—paired t-tests for parametric data and Wilcoxon signed-rank tests for non-normal distributions. To identify predictors of end-stage renal disease (ESRD), we conducted multivariable Cox proportional hazards regression incorporating clinically relevant covariates. Renal survival was visualized through Kaplan-Meier estimates, with between-group differences assessed via stratified log-rank testing. All analyses adhered to a two-tailed α-level of 0.05 for significance determination.

## 3 Results

### 3.1 Clinical characteristics

**Figure 1:**
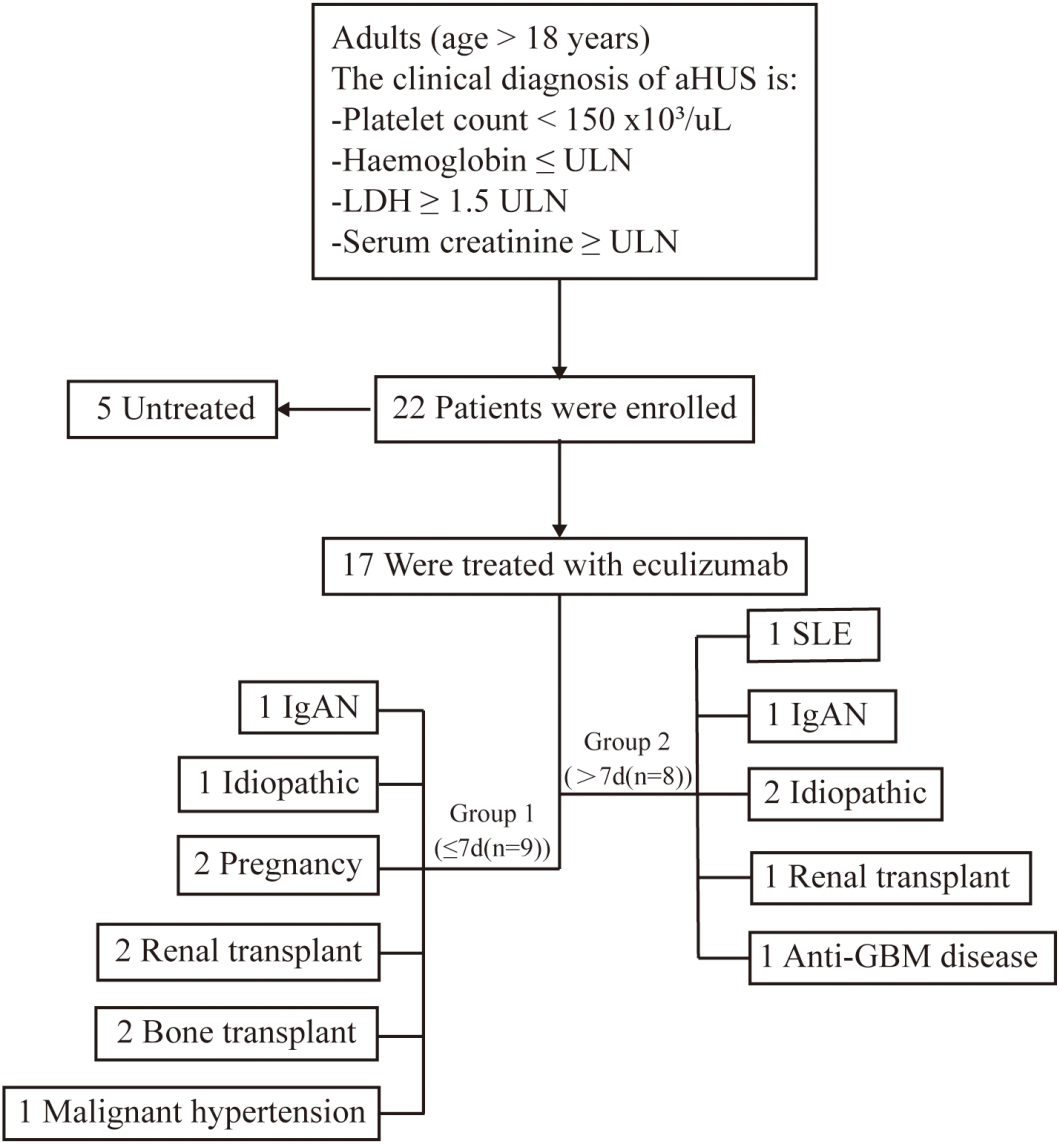
Research flowchart. This study encompassed 17 patients with atypical Hemolytic Uremic Syndrome (aHUS) who were administered eculizumab. aHUS, atypical hemolytic uremic syndrome; ULN, upper limit of the normal range. IgA,immunoglobulin A nephritis; GBM,anti-Glomerular Basement Membrane nephritis; SLE, Systemic Lupus Erythematosus.

The cohort comprised 17 aHUS patients (76.5% female; mean age 43.2 ± 20.0 years) with diverse etiologies: post-pregnancy (n=2), post-transplant (bone marrow: n=2; kidney: n=3), autoimmune (anti-GBM: n=1; lupus nephritis: n=1), malignant hypertension (n=1), and idiopathic (n=7). Genetic testing (n=7) identified a pathogenic CFHR3 variant in one pregnancy-associated case. Baseline characteristics revealed severe hematological and renal dysfunction: hemoglobin 72.9 ± 17.3 g/L, platelets 75.4 ± 44.4 × 10^9^/L, serum creatinine 647.0 μmol/L (IQR 439.0–915.0), and eGFR 8.0 ml/min/1.73m² (IQR 5.0–11.0). Eleven patients (64.7%) required dialysis initiation, with seven undergoing plasma exchange (**Table 1**). Genetic testing was available in 7 cases, and 1 case (pregnacy-associated case 8) was found CFHR3 genetic variates. Seven patients (41.2%) received plasma exchange (median 2 sessions; range 1-4) before eculizumab initiation. Hemodialysis was required in 64.7% (n=11) at baseline, declining significantly to 41.2% (n=7) by final follow-up (**Figure 1**).

**Table 1:** Baseline characteristics stratified by treatment timing.

Variables	Total (n = 17)	Group 1 (≤7d, n = 9)	Group 2 (>7d,n = 8)	P
Age, Mean ± SD	43.2 ± 20.0	46.0 ± 20.3	40.1 ± 20.6	0.563
Sex, n (%)				0.029
male	4 (23.5)	0 (0)	4 (50)	
female	13 (76.5)	9 (100)	4 (50)	
Follow-up time (mons)	11.5 ± 3.5	11.2 ± 2.5	11.8 ± 4.6	0.77
Time from onset to initiation of Eculizumab treatment (d)	15.0 (7.0, 30.0)	7.0 (6.0, 7.0)	30.0 (30.0, 60.0)	< 0.001
Eculizumab treatment course(times)	9.2 ± 4.5	8.7 ± 1.7	9.8 ± 6.5	0.634
PE, n (%)	7 (41.2)	3 (33.3)	4 (50)	0.637
Renal biopsy, n (%)	8 (47.1)	5 (55.6)	3 (37.5)	0.637
Creatinie (umol/L)	647.0 (439.0, 915.0)	561.0 (257.0, 857.0)	682.5 (505.0, 942.5)	0.386
eGFR(ml/min/1.73m2)	8.0 (5.0, 11.0)	8.0 (6.0, 22.0)	6.8 (4.8, 9.5)	0.385
LDH (U/L)	411.0 (382.0, 613.0)	613.0 (408.0, 1214.0)	395.0 (356.8, 427.2)	0.124
Platelet (×10^9/L)	75.4 ± 44.4	76.6 ± 49.7	74.1 ± 41.0	0.915
Hemoglobin (g/L)	72.9 ± 17.3	77.7 ± 15.1	67.6 ± 19.1	0.246
Renal remission, n (%)	9 (52.9)	8 (88.9)	1 (12.5)	0.003
Hematological remission, n (%)	10 (58.8)	9 (100)	1 (12.5)	< 0.001
CKD stage, n (%)				0.001
1	2 (11.8)	1 (11.1)	1 (12.5)	
2	6 (35.3)	6 (66.7)	0 (0)	
4	2 (11.8)	2 (22.2)	0 (0)	
5	7 (41.2)	0 (0)	7 (87.5)	
Need of dialysis at onset, n (%)	11 (64.7)	5 (55.6)	6 (75)	0.62
Dialysis-dependent at follow-up (n, %)	7 (41.2)	0 (0)	7 (87.5)	< 0.001

PE, Plasmapheresis.

Patients were stratified by symptom-to-eculizumab interval: Group 1 (≤7 days; n=9) were all female with median treatment initiation at 7 days (IQR 6-7), while Group 2 (>7 days; n=8) included 50% males with significantly delayed median initiation at 30 days (IQR 30-60; p<0.001), with no significant between-group differences in age, baseline laboratory parameters, or dialysis requirement at disease onset (all p>0.05) (**Table 1**).

### 3.2 Renal pathology features

**Figure 2:**
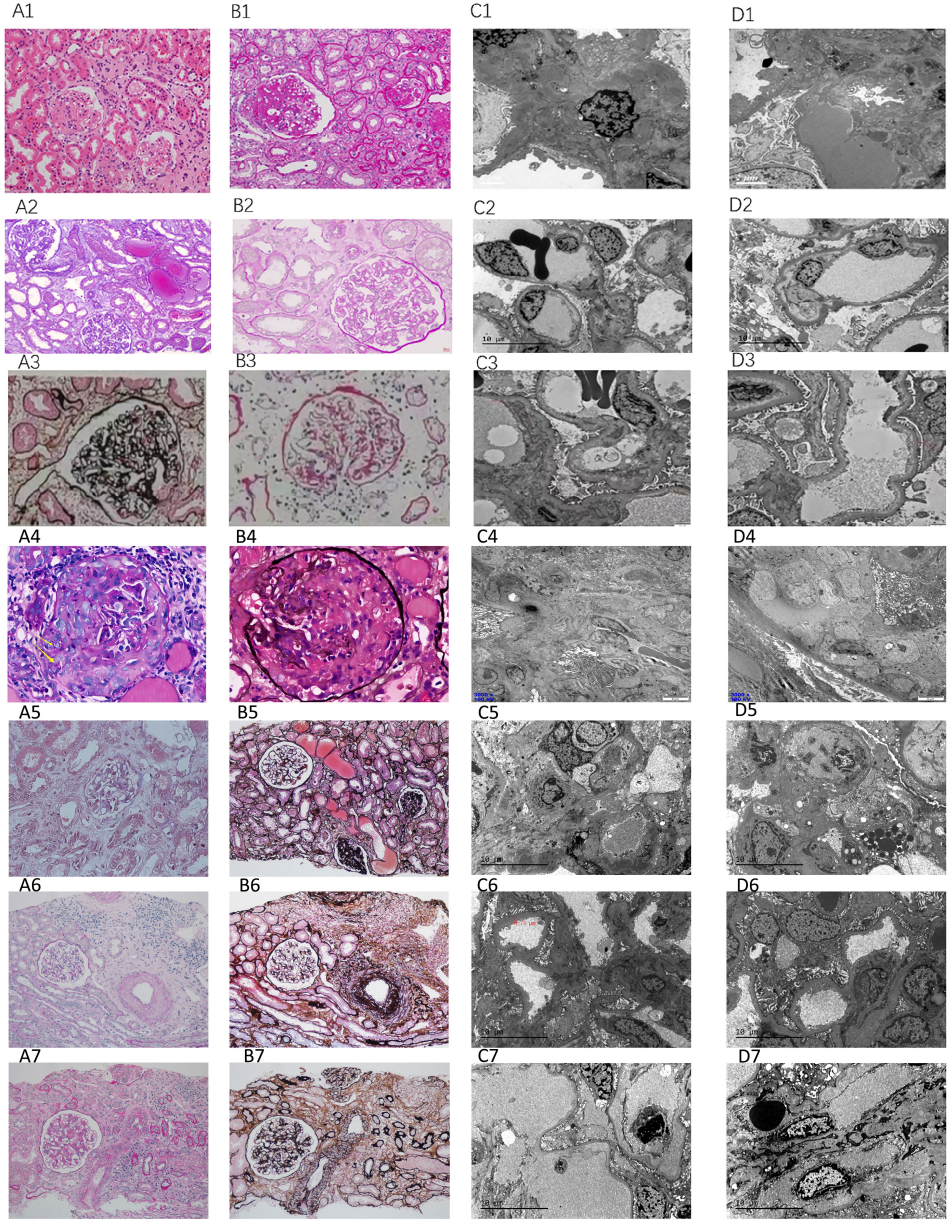
Pathological features. Case 1: Acute tubular injury (**A1**: HE, ×200), diffuse interstitial inflammatory cell infiltration (**B1**: PAS, ×200), arteriolar hyaline degeneration, and endarteritis; under electron microscopy, the basement membrane of the capillary loop is subepithelial and loose (**C1**: EM, ×1600), and 30% fusion of podocyte foot processes (**D1**: EM, ×1600); Case 2: Vacuolation and granular degeneration of renal tubular epithelial cells, focal tubular atrophy and interstitial fibrosis, multifocal flattening of renal tubular epithelial cells, loss of brush border or epithelial cell shedding with partial dilation of the tubular lumen, visible “naked basement membrane” formation (**A2**: Masson, ×400), focal interstitial lymphocyte, monocyte, and a small number of plasma cell infiltration (20%), thickening of the arteriolar wall with mild swelling of endothelial cells (**B2**: PAS, ×400); under electron microscopy, segmental endothelial proliferation (**C2**: EM, ×1600), and widening of the subepithelial loose layer (**D2**: EM, ×1600); Case 5: Global glomerularsclerosis (**A3**: PASM, ×400), segmental sclerosis with adhesion of the glomerular capsule, mesangial proliferation, and tubular atrophy (**B3**: PAS, ×400); under electron microscopy, segmental glomerular sclerosis (**C3**: EM, ×1600), and electron-dense deposits in the mesangial area (**D3**: EM, ×1600).Case 6: Cellular fibrous crescents (**A4**: PAS ×400), rupture of the basement membrane of Bowman’s capsule (**B4**: Hexaamine silver, ×400), segmental wrinkling of the basement membrane (**C4**: EM, ×3000), interstitial inflammatory cell infiltration with fibrosis (**D4**: EM, ×3000);Case 8: Global sclerosis, segmental sclerosis (**A5**: Masson, ×200), multifocal detachment of the brush border or epithelial cells with tubular lumen expansion (**B5**: PASM, ×200), electron-dense deposits in the mesangial area and subendothelial space (**C5**: EM, ×1600), fusion of podocyte foot processes, vacuolar degeneration in the capillaries (**D5**: EM, ×1600);Case 10: Acute tubular injury (**A6**: PAS, ×200), interstitial inflammatory cell infiltration (**B6**: PASM, ×200), subepithelial loosening of the basement membrane of the capillary loop (**C6**: EM, ×1600), fusion of podocyte foot processes (**D6**: EM, ×1600);Case 13: Mesangial proliferative glomerulonephritis (**A7**: PAS, ×200), interstitial inflammatory cell infiltration (**B7**: PASM, ×200), subepithelial loosening of the basement membrane of the capillary loop (**C7**: EM, ×1600), thickening of the subepithelial space of the basement membrane (**D7**: EM, ×1600).

Pathological examination was performed in cases 1, 2, 5, 6, 8, 10, 13 with renal biopsies. Renal histopathology in our biopsy cohort (n=7) confirmed TMA through canonical features: endothelial cell injury (subendothelial widening in 86% of cases), microvascular thrombosis (43%), and ischemic parenchymal damage. Notably, two distinct patterns emerged: (i) complement-dominant TMA with minimal immune deposition (71.4%), and (ii) immune complex-associated TMA with IgA/IgG co-deposition (Cases 5–6), suggesting possible secondary triggers. This aligns with global data where ~30% of aHUS presents with overlapping autoimmune or glomerulopathies. The severity of histological lesions—particularly crescents in Case 6 and advanced sclerosis in Case 5—corroborated clinical outcomes, underscoring the prognostic value of biopsy in complex aHUS presentations (**Table 2**) (**Figure 2**).

**Table 2:** Pathological features of 7 patients diagnosed as aHUS.

	IF (Immunofluorescence)					LM (Light microscopy)						EM(Electron microscopy)		
	IgA	IgM	IgG	C3	Deposition site	Global glomerulosclerosis %	Segmental sclerosis%	Crescents%	Microthrombi	Tubular atrophy/interstitial fibrosis	Inflammatory cell infiltration	Electron-dense deposit	Foot process effa cement	Endothelial cell lamination wid ening
case1	0	0	0	0	Capillary Loops	0	0	0	0	1	2	0	0	1
case2	0	0	0	0		5.3	0	0	1	1	2	0	1	1
case5	1	1	0	1	Mesangial Area	20	46.7	20	0	2	1	0	0	1
case6	0	0	1	1	Capillary Loops	4.8	0	90.5	0	1	3	1	1	1
case8	0	1	0	1		18.2	9.1	0	0	1	1	0	0	1
case10	0	0	0	1	Mesangial Area	0	0	0	0	1	1	0	0	1
case12	0	0	0	0		0	0	0	1	1	1	0	0	1

### 3.3 Efficacy analysis

**Figure 3:**
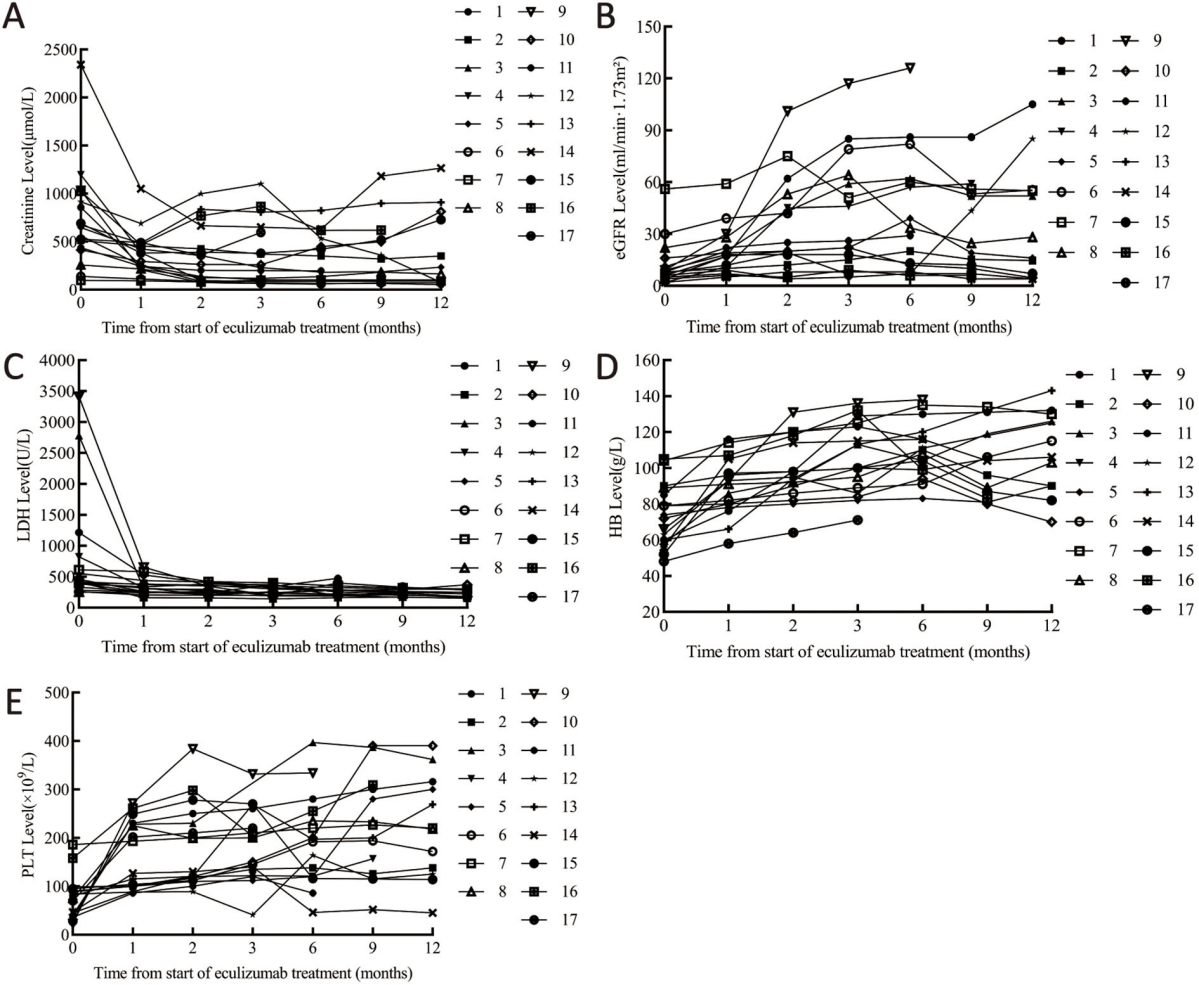
Laboratory parameter changes pre/post eculizumab. **(A)** Changes in creatinine. **(B)** Changes in eGFR. **(C)** Changes in LDH. **(D)** Changes in platelets. **(E)** Changes in hemoglobin. Cr, creatinine; eGFR, Estimated Glomerular Filtration Rate; LDH, lactate dehydrogenase; PLT, platelet; Hb, Hemoglobin.

The median interval from symptom onset to initiation of eculizumab treatment was 15.0 (7.0, 30.0) days, with patients receiving eculizumab treatment ranging from 9.2 ± 4.5 doses. Quantitative evolution of biomarkers confirmed rapid eculizumab efficacy: creatinine decreased >50% within 1 month (Median (IQR)382.0 (229.0, 475.0) μmol/L), with sustained improvement to 198.5 (86.5, 749.5) μmol/L at 12 months. Platelets normalized (≥150 × 10^9^/L) by 1 months (162.9 ± 70.7 × 10^9^/L), while eGFR doubled by 1 months (17.0 (10.0, 22.0) mL/min/1.73m²), though final values remained impaired (21.6 ± 10.3 mL/min/1.73m²) reflecting baseline injury severity (**Table 3**, **Figure 3**).

**Table 3:** Evolution of hematological and renal parameters following eculizumab initiation.

Laboratory indicators	Baseline	Change in time point of eculizumab treatment					
		1 month	2 month	3 month	6 month	9 month	12 month
CR, Median (IQR)	647.0 (439.0, 915.0)	382.0 (229.0, 475.0)	263.0 (95.0, 426.0)	230.0 (99.0, 595.0)	189.5 (96.8, 470.5)	253.5 (93.0, 513.5)	198.5 (86.5, 749.5)
GFR, Median (IQR)	8.0 (5.0, 11.0)	17.0 (10.0, 22.0)	20.0 (12.0, 45.0)	22.0 (9.0, 59.0)	31.3 (11.0, 60.5)	21.9 (10.5, 52.9)	22.1 (6.4, 55.1)
LDH, Mean ± SD	787.5± 908.0	345.1 ± 135.9	291.9 ± 85.0	267.5 ± 78.5	267.6 ± 86.3	245.1 ± 51.1	232.8 ± 70.0
Plt, Mean ± SD	75.4 ± 44.4	162.9 ± 70.7	180.4 ± 85.0	183.1 ± 75.5	193.8 ± 92.7	220.4 ± 103.4	222.4 ± 107.4
Hb, Mean ± SD	72.9 ± 17.3	89.3 ± 16.0	98.4 ± 17.2	105.5 ± 19.6	109.9 ± 15.6	104.6 ± 19.5	109.3 ± 22.8

Cr, creatinine; eGFR, Estimated Glomerular Filtration Rate; LDH, lactate dehydrogenase; PLT, platelet; Hb, Hemoglobin.

Significant outcome disparities emerged between groups: Group 1 demonstrated markedly higher renal remission (88.9% vs 12.5%, p=0.003) and hematological remission rates (100% vs 12.5%, p<0.001), Group 2 exhibited significantly worse CKD progression with 87.5% reaching stage 5 versus none in Group 1 (p=0.001), resulting in substantially higher dialysis dependence at follow-up (0% vs 87.5%, p<0.001).

**Figure 4:**
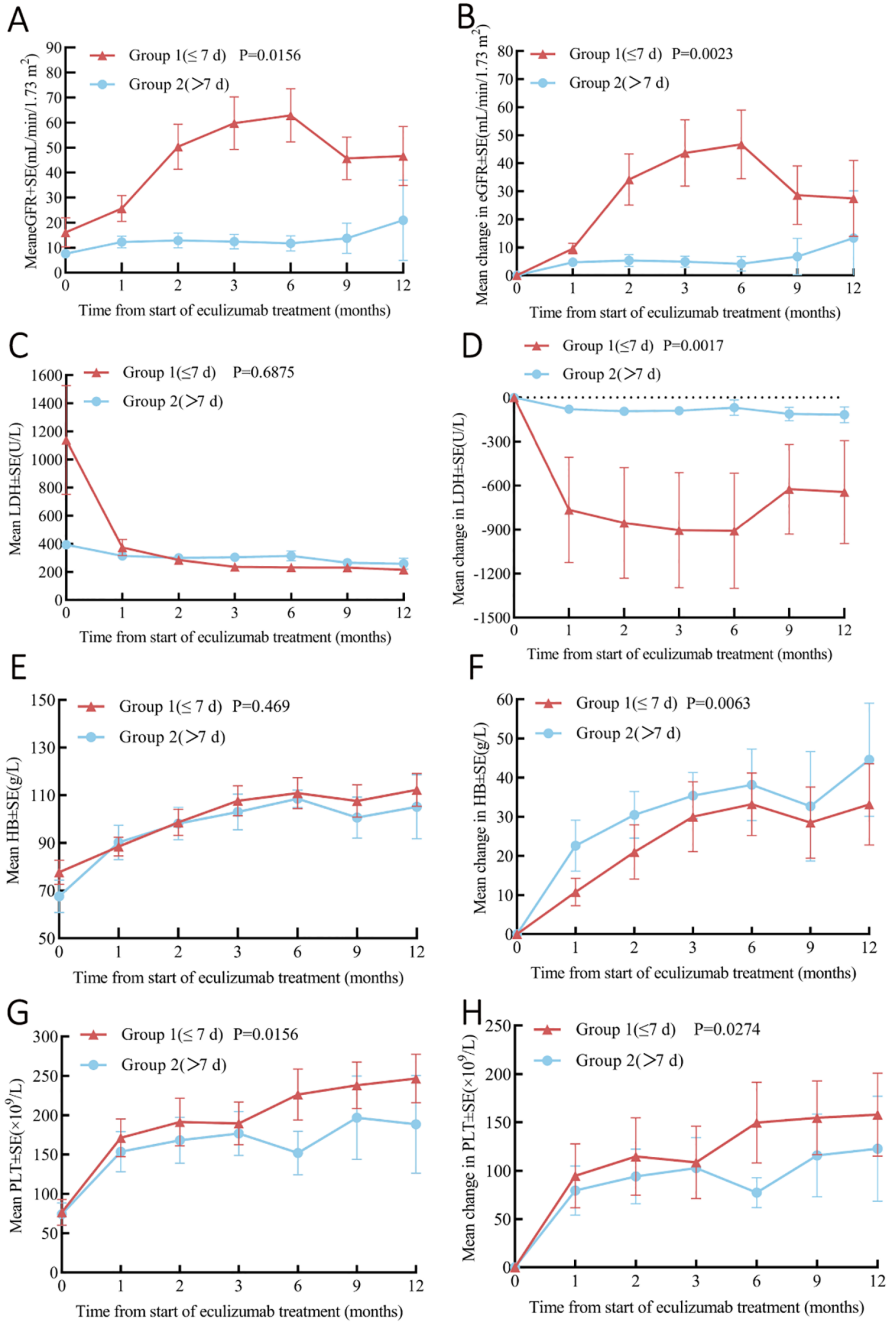
Analysis of Pre- and Post-Eculizumab Treatment Laboratory Parameters Stratified by Time to Eculizumab Administration: Group 1 (≤7 Days, n=8) and Group 2 (>7 Days, n=8). **(A)** Change in eGFR. **(B)** Change in eGFR from baseline. **(C)** Change in LDH. **(D)** Change in LDH from baseline. **(E)** Change in platelets. **(F)** Change in platelets from baseline. **(G)** Change in hemoglobin. **(H)** Change in hemoglobin from baseline. Cr, creatinine; eGFR, Estimated Glomerular Filtration Rate; LDH, lactate dehydrogenase; PLT, platelet; Hb, Hemoglobin.

Stratification by treatment timing revealed significantly greater improvements in Group 1 (≤7 days) versus Group 2 (>7 days): ΔeGFR +19.5 ± 3.1 vs. +2.3 ± 1.7 mL/min/1.73m² (P=0.016), ΔLDH -412 ± 98 vs. -68 ± 42 U/L (P=0.002), Δhemoglobin +35.2 ± 2.9 vs. +8.1 ± 3.1 g/L (P=0.017), and Δplatelets +67.8 ± 9.8 vs. +19.3 ± 7.2 × 10^9^/L (P=0.007), demonstrating superior therapeutic efficacy with early intervention (**Figure 4**).

### 3.4 Predictors for ESRD

**Figure 5:**
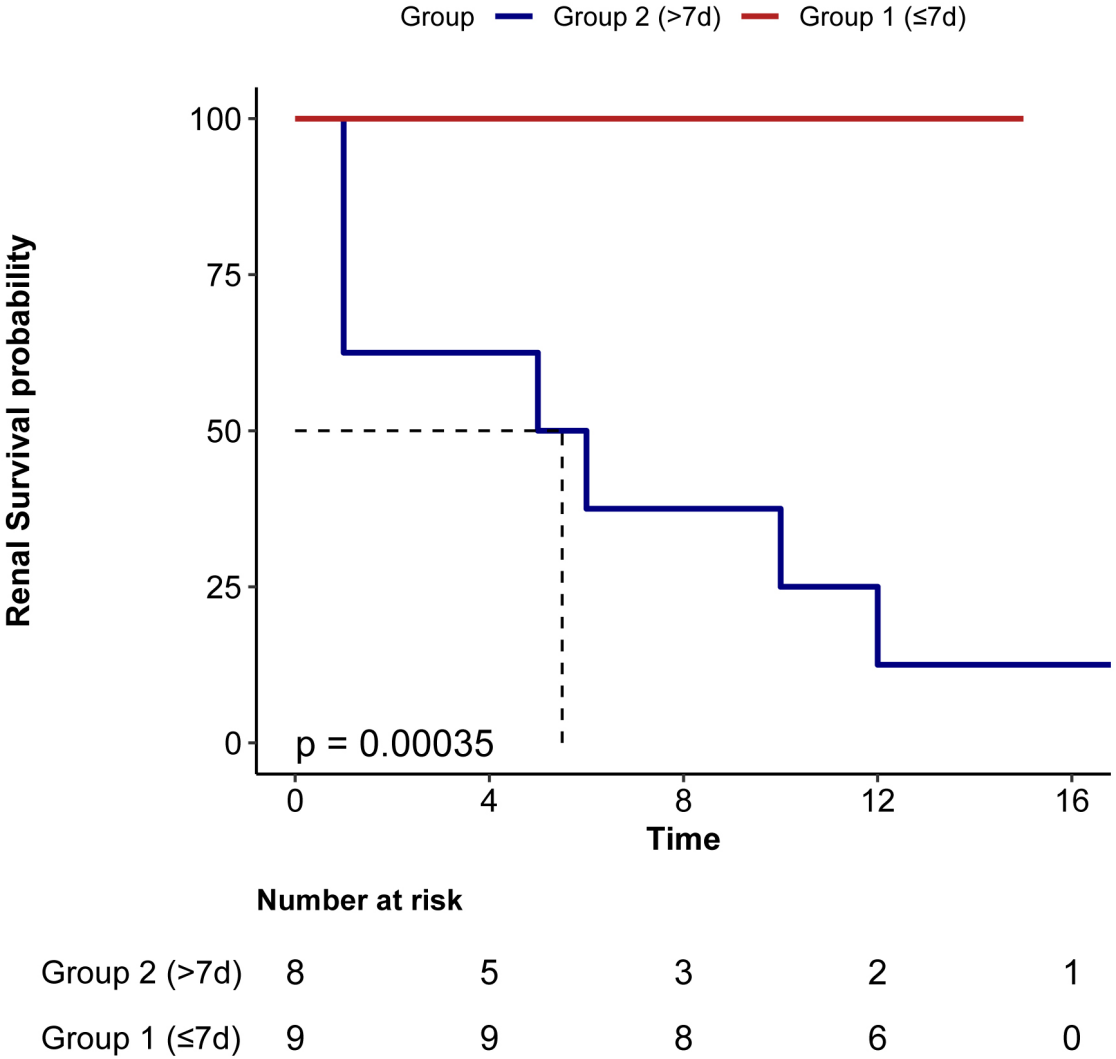
Renal survival in patients based on the Interval from symptom onset to eculizumab initiation. Group 1 (≤7d) had significant better renal survival than group 2 (>7d).

Univariate Cox regression analysis indicated that the time from Symptom Onset to Eculizumab Initiation (HR 1.02, P=0.038) and Creatinine (HR 1.00014, P=0.043) were risk factors for ESRD in aHUS patients. Multivariate Cox regression analysis indicated that only time from Symptom Onset to Eculizumab Initiation (HR 1.02, P-0.038) was independently associated with ESRD events. Kaplan-Meier analysis showed significantly higher renal survival in Group 1 (≤7 days) versus Group 2 (>7 days) (log-rank P<0.001)(**Table 4**, **Figure 5**).

**Table 4:** Association between time-to-eculizumab and ESRD risk .

Characterisitic	OR	Model 1			Model 2			Model 3	
		95%CI	P-value	OR	95%CI	P-value	OR	95%CI	P-value
Time to Eculizamab	1.02	(1.01~1.04)	0.003	1.02	(1~1.03)	0.05	1.01	(0.95~1.05)	0.905

OR, odds ratio; CI, confidence intervals;

The model 1 was the crude model.

The model 2 was adjusted by Age, Sex.

The model 3 was adjusted by Age, Sex, eGFR and dialysis.

Time to Eculizamab: time from Symptom Onset to Eculizumab Initiation; LDH, lactate dehydrogenase; PLT, platelet; Hb, Hemoglobin.

### 3.5 Safety

In the course of this study, two patients experienced adverse events. One patient developed a bloodstream infection caused by Achromobacter xylosoxidans, and another patient experienced a bloodstream infection due to Pseudomonas aeruginosa. Both infections were managed with appropriate symptomatic and antimicrobial treatments, leading to resolution of the infections. Throughout the study, there were no serious adverse events or treatment-related mortalities observed.No meningococcal infections were reported.

## 4 Discussion

This study addresses atypical hemolytic uremic syndrome (aHUS), a rare but severe disorder characterized by microangiopathic hemolytic anemia, thrombocytopenia, and acute renal failure (13). Current management strategies have evolved significantly, with eculizumab—a monoclonal antibody that inhibits the complement component C5—emerging as a prominent therapeutic option that has demonstrated efficacy in halting the progression of renal damage in aHUS patients (14–19). While global evidence supports eculizumab’s efficacy and safety in atypical hemolytic uremic syndrome (aHUS), its high cost (∼¥500,000/US$70,000 monthly) limited clinical use in China until inclusion in the National Reimbursement Drug List (NRDL) in 2024. This access barrier contributed to scarce real-world evidence from Chinese populations. Our study reports the largest single-center cohort (n=17) of eculizumab-treated aHUS patients in mainland China, addressing this knowledge gap.

Although NRDL coverage represents significant progress, sustainability concerns persist—including hospital budget constraints and substantial patient copayments. These economic pressures create treatment dilemmas when considering extended therapy duration, particularly for high-risk patients with genetic variants or suboptimal renal recovery. Rigorous cost-effectiveness studies are urgently needed to guide optimal treatment strategies.

This single-center retrospective analysis of 17 aHUS patients from Western China highlights the transformative impact of early complement blockade. Larger collaborative studies are needed to determine whether trigger-specific dosing or duration adjustments could optimize outcomes. Initiating eculizumab within 7 days of symptom onset—a benchmark achievable in only 53% of cases due to regional diagnostic delays—was associated with 100% hematological remission and reduced dialysis dependency, underscoring urgent needs for healthcare infrastructure optimization. Timely intervention correlated with superior renal recovery, reduced dialysis dependency, and higher rates of hematological stabilization, aligning with global evidence on complement blockade efficacy in aHUS, consistent with prior studies that earlier intervention correlates with improved prognostic outcomes (11, 20–22), emphasizing the importance of the treatment timeline. These findings not only support the hypothesis of the positive impact of prompt treatment but also aim to guide clinical practices in managing aHUS more effectively (23–25).

The present study offers a unique insight into the etiologies of atypical Hemolytic Uremic Syndrome (aHUS) within a Chinese cohort, highlighting the diversity of causes and the complexity of this rare disease. The heterogeneity of triggers in our cohort—including pregnancy, transplantation, and autoimmune disorders—highlights that aHUS often arises within complex clinical contexts, which is consistent with global patterns where these conditions are known to precipitate aHUS (26). While evidence suggests secondary forms may exhibit modified complement activation profiles (27), emergent C5 inhibition remains the cornerstone of TMA management regardless of etiology (28–30). However, the presence of anti-GBM nephritis and lupus nephritis as etiologies in our study is noteworthy and suggests a potential overlap with other autoimmune diseases, which may warrant further investigation into the autoimmune basis of aHUS.

Treatment duration in our cohort was individualized, balancing relapse risks against China’s recent eculizumab reimbursement policies, which prioritize cost-effective short-term regimens (31). Our study, which individualized treatment duration based on patient response and clinical course, aligns with the current trend in the literature, which emphasizes the need for personalized management strategies. Most of our cohort received at least three months of eculizumab, the variability in treatment duration is influenced by several factors, including the presence of underlying genetic mutations, complications following renal transplantation, and the potential risks associated with long-term complement inhibition. Our findings echo those of Fakhouri et al, who in a prospective phase 4 study (32), suggested that discontinuing eculizumab is safe in most patients with aHUS once they achieve complete remission, with a relapse risk of less than 25% overall, though this risk escalates to as high as 50% in patients with rare variants in complement genes (33).The decision to discontinue eculizumab must be carefully considered, as it is influenced by the balance between the high cost and infusion burden of lifelong treatment, potential side-effects, and the risk of relapse (34). This underscores the importance of a comprehensive assessment of each patient’s genetic background, trigger factors, and risk of relapse, as well as the availability of funding for re-treatment and the patient’s ability to adhere to rigorous monitoring protocols.The risk of relapse post-discontinuation is a significant consideration, and close monitoring is required to ensure rapid re-initiation of treatment at the earliest signs of TMA. Future studies should explore biomarkers (e.g., complement activation markers) to guide personalized therapy within resource-limited settings.

Adverse events associated with eculizumab therapy are relatively rare (8). The most common adverse event is infusion-related reactions, which can be managed with symptomatic treatment. However, in our findings, one patient developed a bloodstream infection caused by Achromobacter xylosoxidans, and another experienced a bloodstream infection due to Pseudomonas aeruginosa. The occurrence of bloodstream infections in patients treated with eculizumab is not unprecedented and has been previously reported in the literature. Inhibition of the complement system also comes with an increased risk of infections, as the complement system plays a crucial role in immune defense against pathogens (35).The management of these infections aligns with the standard of care recommended in the literature, emphasizing the importance of prompt diagnosis and targeted antimicrobial therapy.

Several limitations warrant discussion. First, the relatively small sample size and single center study may limit the generalizability of our findings and the ability to detect subtle differences in treatment response among diverse patient populations. Second, despite employing PLASMIC scores for initial risk stratification when ADAMTS-13 activity was unavailable and subsequently excluding patients with ADAMTS-13 activity <10%, rare cases of TTP with atypical presentations could theoretically remain undetected. While the prompt availability of ADAMTS-13 results (within 2-5 days for all patients) significantly strengthened our exclusion criteria, the retrospective design limits causal attribution and introduces potential selection biasrelated to data collection and interpretation, as with any retrospective study design. Third, absent genetic profiling precludes analysis of mutation-specific responses-particularly relevant given the 50% relapse risk in patients with complement gene variants. Future research should aim to include larger, multi-center trials with long-term follow-up to validate our findings and explore the long-term effects of early intervention in aHUS.

In summary, this study evaluates the therapeutic effect and safety of eculizumab in patients with aHUS in China, emphasizing the critical aspect of timely treatment initiation. The findings reinforce the need for prompt diagnosis and intervention to optimize patient outcomes and mitigate the risk of long-term complications associated with delayed treatment. Future research should focus on larger cohorts and extended follow-up to better understand the long-term benefits and applicability of eculizumab in diverse populations.

## Data Availability

The original contributions presented in the study are included in the article/supplementary material. Further inquiries can be directed to the corresponding author.
